# Sexual Exposure to HIV Infection during the COVID-19 Pandemic in Men Who Have Sex with Men (MSM): A Multicentric Study

**DOI:** 10.3390/ijerph18189584

**Published:** 2021-09-11

**Authors:** Alvaro Francisco Lopes de Sousa, Shirley Verônica Melo Almeida Lima, João Victor Rocha, Herica Emilia Félix de Carvalho, Artur Acelino Francisco Luz Nunes Queiroz, Guilherme Schneider, Layze Braz de Oliveira, Emerson Lucas Silva Camargo, Adélia Dalva da Silva Oliveira, Isabel Amélia Costa Mendes, Inês Fronteira

**Affiliations:** 1 Human Exposome and Infectious Diseases Network, Centro Universitário UNINOVAFAPI/AFYA, Piauí 64073-505, Brazil; adelia.oliveira@uninovafapi.edu.br; 2 Global Health and Tropical Medicine, Instituto de Higiene e Medicina Tropical, Universidade Nova de Lisboa, 1349-008 Lisboa, Portugal; ifronteira@ihmt.unl.pt; 3 Núcleo de Investigação em Saúde Coletiva, Universidade Federal de Sergipe, Aracaju 49100-000, Brazil; shirleymelo.lima@gmail.com; 4 Escola Nacional de Saúde Pública, Universidade Nova de Lisboa, 1349-008 Lisboa, Portugal; joao096victor@gmail.com; 5 Human Exposome and Infectious Diseases Network, Escola de Enfermagem de Ribeirão Preto, Universidade de São Paulo, Ribeirão Preto 14040-902, Brazil; hericacarvalho@usp.br (H.E.F.d.C.); arturqueiroz@usp.br (A.A.F.L.N.Q.); guilherme.schneider@usp.br (G.S.); layzebraz@gmail.com (L.B.d.O.); lucmrg0@gmail.com (E.L.S.C.); iamendes@eerp.usp.br (I.A.C.M.)

**Keywords:** sexual exposure, HIV/AIDS, COVID-19, SARS-CoV-2, sexual behavior, men who have sex with men

## Abstract

The practice of sex with casual partners without the use of adequate prevention in the period of social distancing due to the COVID-19 pandemic among men who have sex with men (MSM) can expose them to the risk of infection by the HIV virus. To assess this, we conducted an online survey in April and May 2020 in the entire national territory of Brazil and Portugal. We used the snowball technique for sampling, associated with circulation in social networks, totaling 2934 participants. Bivariate and multivariate logistic regression was used to produce the adjusted Odds Ratio (aOR). Eight-hundred-and-forty-two (28.7%) MSM presented at-risk sexual exposure in this period. In general, the types of sexual practices that most increased the chances of sexual exposure were having multiple partners (aOR:14.045); having practiced chemsex (aOR:2.246) and group sex (aOR:2.431), as well as presenting a history of at-risk sexual exposure (aOR:5.136). When we consider each country separately, the chances are increased in Brazil since the probability of the outcome was increased in those who practiced group sex (aOR:5.928), had multiple partners (aOR:19.132), and reported a sexual history of at-risk exposure (aOR:8.861). Our findings indicate that practices that are classically associated with greater chances of engaging in risky sexual exposure to HIV infection were the factors that most increased the chances of acquiring the virus in the pandemic context.

## 1. Introduction

The World Health Organization (WHO) declared the outbreak of COVID-19, an infectious disease caused by the new SARS-CoV-2 coronavirus, as the sixth public health emergency of international concern on 30 January 2020, and as a pandemic on 11 March 2020 [1,2], when the pandemic, which started in Wuhan, China, had already spread to 114 countries, totaling 118,319 cases and 4292 deaths due to the disease [3].

In Brazil, the first COVID-19 case was confirmed on 26 February 2020 [4] and, in Portugal, on 2 March 2020 [5]. Due to the scarcity of information about the new virus and the absence of specific and effective vaccines and antivirals for the prevention and treatment of the disease [6,7], the response to the COVID-19 pandemic at the local and global levels was initially grounded on non-pharmacological interventions that included, among others, social distancing and isolation measures, isolation of patients and screening of their contacts, and lockdown [8,9,10]. Previous epidemics showed that, while the interventions remained in force, mortality was reduced but, when they were withdrawn or relaxed, transmission was increased [11].

Brazil and Portugal, countries where the same language is spoken and which share a large number of immigrants, experienced these epidemiological moments differently throughout 2020: in April and May 2020 (study period), Brazil was in the mitigation phase [12,13], while Portugal was in the suppression phase [14]. In this context, the social distancing adopted in these two countries, and worldwide, provided a reduction in the infection transmission rates, but generated impacts on the mental and sexual health of the individuals, especially in the LGBTQIA+ population [15,16,17,18,19]. Among the individuals in this population, Men who have Sex with Men (MSM) present difficulties in following the restrictive measures, especially those related to the affective and sexual practices [16,17,18,19,20].

Despite the risk of infection by SARS-CoV-2, MSM living in Brazil and Portugal still continued having sex with casual partners outside their safe environment, even in the period of social distancing [17] and increased the number of casual sexual partners during the COVID-19 pandemic [21,22]. The behavior of the MSM also recorded an increase in the consumption of alcohol and other drugs [23], in addition to the practice of chemsex (chemical sex or sex under the influence of drugs) [19,24], considerably increasing exposure to HIV. Consequently, it is observed that, in addition to exposure to infection by SARS-CoV-2, MSM are exposed to Sexually Transmitted Infections (STIs), mainly HIV/aids.

In this scenario, we evidenced a possible syndemic [25] when we noticed the synergy between the advance of STIs with the COVID-19 pandemic, producing an increase in the burden of diseases in a vulnerable population [26]. Therefore, understanding how this phenomenon occurs in specific groups is fundamental to ground adequate preventive strategies, aiming to reduce this burden in the health services and in the general population. Studies on interactions, impacts and synergy between the COVID-19 spectrum and at-risk sexual exposure to HIV infection among MSM need to be strengthened and deserve scientific deepening in the search of more details about the behavior of these individuals and of better living and health conditions.

Consequently, we aimed at analyzing factors associated with at-risk sexual exposure to HIV infection during the COVID-19 pandemic in men who have sex with men among residents of Brazil and Portugal.

## 2. Materials and Methods

This is data from the 40TENA (acronym for Quarantine) project, a subproject of the “In_PrEP” multicentric cohort led by Universidade Nova de Lisboa, Portugal in partnership with Universidade de São Paulo, Brazil. The 40TENA project is an online behavioral survey applied in all the 26 Brazilian states and in 15 of the 18 Portuguese districts.

### 2.1. Population, Sampling, and Recruitment

The data collected refer to the period between April and May 2020, when Brazil and Portugal were experiencing restrictive sanitary measures that indicated social distancing and isolation, in addition to quarantine in the positive and symptomatic cases to prevent the virus from spreading. Up to that moment, the complete closure of essential services such as supermarkets and pharmacies (called lockdown) had not yet been implemented. A simple sample calculation for proportion was performed considering 95% confidence level, assumed prevalence of 50%, tolerable error of 2% and a loss replacement rate of around 20%, obtaining 2880 necessary participants.

Due to the COVID-19 pandemic, we recruited MSM from both countries online, using a version of the snowball sampling technique [27] adapted to the online environment. In this method, the participants themselves are responsible for recruiting other individuals in similar situations by means of their social networks and contacts. According to the method’s criteria, we selected 15 MSM with different characteristics in each country. The following was used to classify these initial participants (seeds): place of residence (we chose the most populous state/district in each of the countries’ regions), race (Caucasian/non-white), age (young, adult, or older adult), and schooling level (high school, undergraduate or graduate). Each seed received a link to the research and was instructed to invite at least 5 MSM from their social network until a significant sample was obtained within two months. To identify the seeds, we used the time location sampling (TLS) technique, changing the location to have access to the seeds [28,29]. The two most popular geographic location-based dating apps (Grindr^®^ and Hornet^®^) [30] were used, and a direct approach through chat with online users who met the inclusion criteria and sample diversification was implemented. The first individuals who were available online in each one of the two apps and who met the inclusion criteria were included, according to what is recommended in previous studies [28,29].

The researchers also promoted the research on Facebook^®^, targeting the MSM population aged from 18 to 60 years old (age restriction imposed by Facebook), through a fixed post on the research official page (www.facebook.com/taafimdeque, accessed on 30 August 2021), accompanied by an electronic link that granted access to the free and informed consent form and to the research questionnaire. Facebook^®^ was used as an additional resource due to its ability to access people from the inland, which is absolutely necessary in the case of a continental country like Brazil. Only individuals who identified themselves as men (cisgender or transgender) and aged at least 18 years old were included. Tourists and people who did not speak Portuguese were excluded.

In this way, 3947 MSM were eligible for the research, of which 1300 lived in Portugal and 2647 in Brazil. Since our phenomenon of interest was at-risk sexual exposure to the HIV virus, we excluded from the analysis those MSM who made use of pre-exposure prophylaxis (PrEP) as well as HIV positive individuals undergoing treatment (1013 MSM), totaling 2934 participants.

### 2.2. Data Collection Instrument

The data collection instrument was developed by the researchers and validated (face-content) by 3 expert judges/subject matter specialists, as well as by 7 potential participants. It was divided into five sections with 46 questions, mostly of the multiple-choice type, some of which were mandatory to proceed and finish. The questions addressed sociodemographic information (age, schooling, gender identity, housing arrangement, and relationships), mental health issues (self-perception of stress, coping with the pandemic), behaviors adopted in the face of the pandemic (social distancing, protective measures for COVID 19 and adherence to them), sexual activities and behaviors during the period of social distancing and isolation and/or quarantine, as well as during the period slightly prior to the pandemic outbreak (establishment of relationships, type and number of partners, and protective measures for STIs).

The online form was hosted in a website exclusive to data collection and which only allowed one answer per IP (Internet Protocol) (that is to say, only one answer per electronic device), thus avoiding selection biases.

For this study, we defined casual sex as anal sex with a new or unknown partner who was not previously in the same safe environment as the participants, with the following question: “Since social distancing/safeguarding was proposed in your country, did you have sex with a new or unknown partner who is outside your safe environment or did you leave your safe environment to find that partner? At-risk sexual exposure was defined as unprotected anal sex with a casual partner, that is, without using biomedical protection measures, namely: condom, PEP, PrEP and/or partner testing.

Adherence to the social distancing measures was defined as avoiding personal contact with people outside their safe environment for non-essential activities. “Partial” isolation was considered when the individual had direct and recurrent contact with people outside their safe environment, and total isolation when there was no direct contact. The COVID-19 signs and symptoms and other clinical variables were based on the local recommendations (Brazilian and Portuguese Ministries of Health).

### 2.3. Data Analysis

The data were stored in a Microsoft Excel (Windows version 2013, Microsoft Corporation; Redmond, WA, USA) spreadsheet and the analyses were performed in the Statistical Package for the Social Sciences software, version 24.0 (SPSS Inc., Chicago, IL, USA).

The study outcome (dependent variable) was at-risk sexual exposure to HIV infection (insertive anal sex without a condom) with a casual/unknown/new partner during the COVID-19 pandemic. The sociodemographic variables, sexual behavior, and coping with COVID-19 were considered for the independent variables. Initially, descriptive analyses were performed, which included absolute and relative frequencies with associations estimated by Pearson’s Chi-square test, after verification of the test’s eligibility criteria, considering *p* < 0.05.

An evaluation of multicollinearity between the independent variables was carried out considering the parameters of the tolerance coefficients and VIF (Variance Inflation Factor) to proceed with the multivariate analysis. Bivariate and multivariate logistic regression (developed by the stepwise regression method) and Odds Ratio (OR) and adjusted Odds Ratio (aOR) were used to measure the intensity of association between at-risk sexual exposure and associated factors considering their respective 95% confidence intervals. The statistical model was chosen by the best fit and performance of the “Receiver Operating Characteristic” (ROC) curve when assessing the sensitivity, specificity and accuracy of the predictive model. The models identified specific determining factors for at-risk sexual exposure among the MSM, both in Portugal and in Brazil.

### 2.4. Ethical Aspects

The research project obtained ethical approval from the Universidade Nova de Lisboa, Portugal and Universidade de São Paulo, Brazil. Informed consent was obtained from all users online, before proceeding with the questionnaire.

## 3. Results

A total of 2934 MSM were analyzed, of which 842 (28.7%) had at-risk sexual exposure (insertive sex without a condom) to HIV infection with a casual/unknown/new partner. The findings showed important convergences and divergences of factors that are associated between residents in Brazil and Portugal during the COVID-19 pandemic.

The characteristics related to country of residence, age group, schooling level, isolation practice, impacts felt in the pandemic, type and number of partners, cohabitation with a partner, use of dating apps, Facebook and WhatsApp to search for partners, practice of Chemsex and group sex, COVID-19 symptoms in the period and previous history of at-risk sexual exposure at some point in life showed a significant association with the fact of being exposed to at-risk sex or not during the pandemic (Table 1).

When we analyzed the country of residence, we identified that the MSM living in Brazil present higher chances of at-risk sexual exposure (aOR: 1.961) when compared to Portugal. The impacts caused by the pandemic showed that the smaller the impact, the greater the chance of exposure, and that those who are not in isolation present more chances of sexual exposure to the risk (Table 2).

The MSM who reported an increase in the frequency of their sexual practices (aOR: 1.492), multiple partners (aOR: 14.045), with unknown HIV status (aOR: 2.081) and who practiced chemsex (aOR: 2.246) in this pandemic period, present higher chances of at-risk sexual exposure. Regarding the behavior of searching for new partners, not using dating apps for the search represented 1.549 times more chances of at-risk sexual exposure. Those who have already had COVID-19 symptoms at some point during the pandemic are 2.275 times more likely to have at-risk sexual exposure when compared to those who had no symptoms (Table 2).

When we analyzed the MSM living in Brazil, it was identified that there was greater probability of at-risk exposure in the individuals who reported not practicing isolation (OR: 1.356) in relation to those who were in isolation, who felt little impact (aOR: 1.375) of the pandemic in their lives, and who practiced chemsex (aOR: 1.764) and group sex (aOR: 5.928). Those who reported having multiple partners during the pandemic were 19.132 times more likely to be exposed to sexual risk, as well as those who did not undergo an HIV test in the past 12 months and who had a previous history of at-risk sexual exposure presented more risk of exposure (Table 3).

When we analyzed the MSM living in Portugal, in the multivariate model, four predictive characteristics for at-risk sexual exposure were observed among them. Those not living in the metropolitan area (aOR: 5.673) and not living with a partner (aOR: 5.556) presented higher chances of at-risk sexual exposure. Regarding multiple partners (aOR: 17.808) and those who already had COVID-19 symptoms (aOR: 5.416) in this pandemic period, both present more chance of being exposed to sexual risk (Table 4).

## 4. Discussion

The public health measures adopted to contain the advances of the COVID-19 pandemic can favor the maintenance of classic risk behaviors for HIV, as well as the growth of new infections by the virus. The sexual practices identified, the deepening of the distancing between the male population and the health services due to the closure of the sexual health clinics and the need to reorganize the services in the COVID-19 pandemic may have exerted a direct impact on the screening and diagnosis of STIs, masking their incidence data.

Our findings showed that at-risk sexual exposure to HIV infection was present in 30.6% of the residents in Brazil and in 24.4% of the residents in Portugal, high prevalence values when compared to studies who used similar methodology prior to the pandemic [28,30,31]. Particularly, the MSM living in Brazil presented almost twice the chances of at-risk sexual exposure to HIV infection when compared to those living in Portugal. This can be explained by the fact that in Brazil, unlike the Portuguese scenario, there has been total lack of control of the pandemic for more than a year [32], which was also verified at the time of data collection. For this country, even with the start of immunization against COVID-19, the speed at which it is taking place (less than the ideal) can compromise control of the pandemic and favor the emergence of variant strains, especially among the younger age groups with complete absence of immunization. Added to this, there is the instability in the management of the pandemic since its outbreak, which has recently culminated in a judicial inquiry commission against the federal government.

We noticed that practices classically associated with greater chances of engaging in at-risk sexual exposure to HIV infection in MSM, such as having had multiple partners [33], group sex [34], not knowing the partner’s serological status [28] and practicing chemsex [35] were also determining factors when we consider the COVID-19 pandemic scenario, although a reduction in part of these behaviors could be expected following the promotion of social distancing and isolation.

Despite this, multiplicity of partners was the factor that most increased the chance of the outcome, by more than 14 times, when comparing the two countries—and more than 19 times in Brazil and 17 times in Portugal when each country was considered individually. These findings are extremely important to explain how it is possible that the rates of these sexual behaviors and practices remain high, even under the threat of a potentially fatal disease such as COVID-19. In a scenario of stress and prolonged isolation, the search for sex and recreational drugs can occur simultaneously as a way to alleviate the tensions experienced and to anesthetize the distancing conditions, in addition to the harsh reality of the necropolitics experienced by Brazilians [24,36] or even as a plus, since drug use is often associated with group sex to make it more pleasurable or prolonged [37].

Although these practices are already a habitual concern of the public policies, in the COVID-19 context they become more drastic due to the overlapping of vulnerabilities [36] as a possible syndemic [38]. Trying to deal with this scenario requires developing new forms of coping, as many forms of personal contact became unfeasible during the pandemic, such as group activities, parties, gym and family contact. This context increases the uncertainties about the future and safety of the population, especially for LGBTQIA2+ [39,40], historically more affected by lack of social support, inequalities and difficulties in accessing the health services. It is believed that these practices can represent an escape valve from the harmful social effects of the pandemic, modulated by individual behaviors, not exclusive to Brazilian or Portuguese MSM, but at the global level [21].

Regarding the particularities of the MSM in each country, the isolated analyses show that Brazil still presents worrying conditions, and that the way in which Brazilian MSM face the pandemic affects the chances of exposure to HIV, which was nearly 1.3 times higher in the MSM who do not practice social distancing, as well as in those who felt little impact of the pandemic on their lives. In an unexpected scenario, in which their usual social supports (family and friends) have limited, and sometimes precluded contact, the search for a routine health monitoring is in a second place, but does not hinder the search for sexual experiences. In a risk compensation logic [40,41], the already known barriers to accessing health services (fear, stigma, and welcoming) are added to the risk of being exposed to COVID-19, in contrast to the pleasure and release offered by sex in a stressful situation. It is important to point out that failure to comply with the social distancing measures does not exempt MSM from the concern about at-risk exposure to HIV infection, since the behavior is an attempt to reduce the deleterious effects of the pandemic [18,23,42,43].

An important finding already corroborated by previous studies [17,20] is the maintenance of at-risk sexual behaviors during the pandemic at levels similar to periods prior to it. Our data show that the MSM with a previous history of at-risk sexual exposure presented 8 times more chances of repeating it. It is important to notice how this reveals that social distancing did not create these behaviors, but increased them, although new studies of a longitudinal nature can investigate this trend and provide more robust findings. It is therefore necessary to understand how, in pandemic phases, in which access to the health services can be severely affected, such as in Portugal in the months prior to data collection, it is possible to continue guaranteeing this population access to post-exposure prophylaxis (PEP) as well as other risk reduction measures.

An important trend in health crisis periods is the adoption of parallel and more “easy and convenient” strategies to mitigate infection risks. People create mechanisms that allow them to feel safe, without the need to adopt the current standards. Regarding this, a number of studies [17,44] have shown, for example, the adoption of PrEP as the strategy of some MSM to mitigate SARS-CoV-2, which is considered inefficient. In this study, MSM who have already presented COVID-19 symptoms seem to have felt more engaged in practicing unprotected anal sex, probably because of the sense of safety given by their immunological memory. Although durable immunity against secondary COVID-19 disease is a possibility [45], relaxation of the preventive measures and contact is not indicated, considering the possibility of reinfection and new strains/variants.

In addition to understanding how these factors relate to each other and influence the behavior of MSM, this analysis provides data on a new context that must be considered in health interventions and in sexual health counseling [46,47]. Knowing how these practices are taking place is fundamental for health professionals to shape their care and better adapt to the reality of the individuals, in addition to the fact that this scenario is extremely variable, even within a relationship, in which the couple can present different forms of coping (proactive or passive style) and sexual desire.

The individuals’ emotional state must be taken into account in the analysis of at-risk sexual behaviors and, especially, in health and harm reduction interventions. Surveys conducted in social media showed an increase in the activity on dating sites (e.g., OkCupid, PlentyOfFish) and apps (e.g., Tinder or Grindr) even among individuals who are already in some affective relationship. Our data indicate that the role and impact of sexual relations and the maintenance of certain sexual behaviors in the spread of the virus and the outcome of the pandemic cannot be ignored. Despite this, a study carried out in Portugal [19] points to the maintenance of a high partner turnover as an important infection route for the explosion of new cases experienced in the country at the end of 2020, after the phase of relaxation of the distancing measures.

The dissemination of directive messages aimed at sexual health, such as reducing the number of partners, and encouraging combined prevention for STIs and COVID-19 can be effective at this moment. The New York Health Department developed a recommendation on possible forms of sexual practices, varying from safer to riskier [48]; this approach can be adapted considering the data from this study and also taking into account subjective aspects, such as non-monogamous relationships.

There are several limitations in the current study. Cross-sectional studies do not support conclusions regarding causality; that is, longitudinal studies would allow for better evidence of the associations. This would be particularly important to assess whether the restrictions of the pandemic actually generate the behaviors reported in this study, or whether there is simply a maintenance and exacerbation of these behaviors. Furthermore, the survey instrument was only used to conduct sampling during COVID-19 preventative measures and due to the lack of studies addressing the topic in the literature, comparisons had to be made with previous studies that used different instruments. The data were collected online, with self-reported information, being impossible to verify their veracity, and they only represent those with access to Internet. The sample is predominantly white-skinned and highly educated. With a more racially and economically diverse sample, we may expect to observe greater variation in the negative experiences with COVID-19 (in terms of increases in structural vulnerability) [49]. The site that hosts the form is not able to inform how many subjects were reached, only the number of access instances and answers.

## 5. Conclusions

Our findings indicate that practices classically associated with greater chances of engaging in risky sexual exposure to HIV infection were the factors that most increased the chances of MSM becoming infected with the virus in the context of the COVID-19 pandemic. Having multiple partners, increasing sexual frequency, practicing group sex and chemsex, or ignoring the partner’s serological status were the main factors. This is a concerning trend that needs to be studied in more depth. Expanded understanding of these factors, especially in a pandemic situation, requires different response plans to achieve better health conditions. The development of communication strategies tailored for MSM, as well as ensuring access to health care targeted at this population is fundamental to reduce the syndemic risk of two epidemics: SARS-CoV-2 and HIV.

## Figures and Tables

**Table 1:** Characteristics of Men who Have Sex with Men and their relation with exposure or non-exposure to at-risk sexual practices during the COVID-19 pandemic, Brazil and Portugal, 2020.

Factors		Exposure to at-Risk Sexual Practices	Non-Exposure to at-Risk Sexual Practices	*p*-Value
Country of residence	Brazil	623 (30.6)	1413 (69.4)	<0.001 *
	Portugal	219 (24.4)	679 (75.6)	
Age group	18–25 years old	212 (27.9)	547 (72.1)	0.003 *
	26–39 years old	452 (27.2)	1212 (72.8)	
	40+ years old	178 (34.8)	333 (65.2)	
Schooling level	Elementary School	0 (0.0)	0 (0.0)	0.009 *
	High School	13 (20.3)	51 (79.7)	
	Higher Education	105 (27.3)	280 (72.7)	
	Postgraduate Degree	564 (33.4)	1125 (66.6)	
Lives in the Metropolitan Area	Yes	662 (27.9)	1707 (72.1)	0.065
	No	180 (31.9)	385 (68.1)	
In isolation	Yes	580 (26.9)	1573 (73.1)	<0.001 *
	Partially	240 (34.2)	461 (65.8)	
	No	22 (27.5)	58 (72.5)	
How much has the COVID-19 pandemic affected you?	A little	285 (23.7)	920 (76.3)	<0.001 *
	To some extent	475 (35.3)	872 (64.7)	
	Very much	82 (21.5)	300 (78.5)	
Type of partners	Casual	538 (28.4)	1359 (71.6)	<0.001 *
	Stable and occasional	174 (36.6)	301 (63.4)	
	Stable	130 (23.1)	432 (76.9)	
Lives with partner		97 (25.1)	289 (74.9)	0.002 *
Means used to search for partners during the COVID-19 pandemic				
Dating apps		637 (29.9)	1492 (70.1)	0.046 *
Facebook		343 (26.8)	938 (73.2)	0.020 *
Chat groups		45 (18.6)	197 (81.4)	<0.001 *
Bars		66 (27.2)	177 (72.8)	0.518
During the COVID-19 pandemic:				
Practiced group sex		139 (44.1)	176 (55.9)	<0.001 *
Practiced Chemsex		315 (40.6)	461 (59.4)	<0.001 *
Had multiple partners (3 or more)		487 (58.1)	351 (41.9)	<0.001 *
Already presented COVID-19 symptoms		460 (39.3)	711 (60.7)	<0.001 *
Was already tested for COVID-19		86 (26.6)	237 (73.4)	0.383
During the pandemic, the frequency of your sexual practices	Did not change	147 (26.6)	405 (73.4)	0.485
	Was reduced	586 (29.2)	1418 (70.8)	
	Increased	109 (28.8)	269 (71.2)	
During the pandemic, your number of partners	Did not change	402 (30.8)	905 (69.2)	<0.001 *
	Was reduced	324 (22.5)	1116 (77.5)	
	Increased	116 (62.0)	71 (38.0)	
During the pandemic, your alcohol consumption	Did not change	293 (27.9)	758 (72.1)	0.446
	Was reduced	387 (29.9)	908 (70.1)	
	Increased	162 (27.6)	426 (72.4)	
Do you know your HIV status?	Yes	707 (28.1)	1808 (71.9)	0.085
Tested for HIV in the last 12 months	Yes	473 (29.7)	1120 (70.3)	0.194
Previous history of at-risk sexual exposure	Yes	224 (46.5)	258 (53.5)	<0.001 *

* Chi-square *p*-value < 0.05 (statistically significant difference).

**Table 2:** Bivariate and multivariate analysis of predictive factors for at-risk sexual exposure during the COVID-19 pandemic, Brazil and Portugal, 2020.

Factors		OR (95%CI)	*p*-Value	aOR (95%CI)	*p*-Value
Country of residence	Brazil	1.367 (1.143–1.635)	<0.001	1.961 (1.506–2.554)	<0.001
	Portugal	1.00		1.00	
Lives in the Metropolitan Area	Yes	1.206 (0.989–1.470)	0.065	1.344 (1.028–1.757)	0.003
	No	1.00		1.00	
Age group	18–25 years old	1.00			
	26–39 years old	0.962 (0.794–1.166)	0.694		
	40+ years old	1.379 (1.083–1.756)	0.009		
Schooling level	High School	1.00			
	Higher Education	1.471 (0.769–2.815)	0.244		
	Postgraduate Degree	1.967 (1.061–3.646)	0.032		
In isolation	Yes	1.00		1.00	
	Partially	0.972 (0.590–1.603)	0.228	0.773 (0.423–1.413)	0.353
	No	1.373 (0.820–2.297)	0.912	1.340 (1.159–2.248)	<0.001
Impact	A little	1.758 (1.478–2.092)	<0.001	1.767 (1.399–2.232)	<0.001
	To some extent	0.882 (0.668–1.165)	0.378	0.723 (0.487–1.073)	0.108
	Very much	1.00		1.00	
Type of partners	Casual	1.921 (1.465–2.518)	<0.001		
	Stable and occasional	1.316 (1.056–1.639)	0.015		
	Stable	1.00			
Lives with partner	Yes	1.00		1.00	
	No	1.493 (1.164–1.916)	0.002	1.580 (1.146–2.179)	0.005
Uses dating apps	Yes	1.00		1.00	
	No	1.222 (1.004–1.487)	0.046	1.549 (1.169–2.046)	0.002
Uses Facebook to find partners	Yes	1.00			
	No	1.216 (1.031–1.434)	0.020		
Uses WhatsApp groups	Yes	1.00			
	No	1.871 (1.339–2.615)	<0.001		
Goes to bars to find partners	Yes	1.00			
	No	1.102 (0.821–1.481)	0.518		
Does not look for partners during the pandemic	Yes	1.00			
	No	1.444 (1.158–1.800)	<0.001		
During the pandemic, the frequency of your sexual practices	Did not change	1.00		1.00	
	Was reduced	0.896 (0.669–1.199)	0.460	0.816 (0.588–1.132)	0.223
	Increased	1.020 (0.800–1.300)	0.874	1.492 (1.011–2.202)	0.044
During the pandemic, your number of partners	Did not change	1.00			
	Was reduced	0.654 (0.551–0.775)	<0.001		
	Increased	3.678 (2.677–5.054)	<0.001		
Practiced group sex during the pandemic	Yes	2.152 (1.696–2.733)	<0.001	2.431 (1.720–3.437)	<0.001
	No	1.00		1.00	
Practiced chemsex during the pandemic	Yes	2.115 (1.778–2.516)	<0.001	2.246 (1.672–3.017)	<0.001
	No	1.00		1.00	
Had multiple partners (3 or more) during the pandemic	Yes	6.804 (5.692–8.134)	<0.001	14.045 (10.257–19.231)	<0.001
	No	1.00		1.00	
Already presented COVID-19 symptoms	Yes	2.339 (1.987–2.753)	<0.001	2.275 (1.811–2.858)	<0.001
	No	1.00		1.00	
Was already tested for COVID-19	Yes	1.123 (0.865–1.458)			
	No	1.00			
Alcohol consumption during the pandemic	Did not change	1.00			
	Was reduced	1.016 (0.811–1.274)	0.887		
	Increased	1.121 (0.903–1.392)	0.302		
Do you know your HIV status?	Yes	1.00		1.00	
	No	1.216 (0.973–1.519)	0.086	2.081 (1.534–2.825)	<0.001
Tested for HIV in the last 12 months	Yes	1.112 (0.947–1.307)	0.194		
	No	1.00			
During your life, have you already had at-risk sexual exposure?	Yes	2.577 (2.108–3.150)	<0.001	5.136 (3.849–6.854)	<0.001
	No	1.00		1.00	

OR: odds ratio, aOR: adjusted odds ratio, CI: confidence interval, ROC curve: 0.816 (<0.001); sensitivity: 80.2%; specificity: 73.6%, Nagelkerke R square: 0.350.

**Table 3:** Bivariate and multivariate analysis considering the factors associated with at-risk sexual exposure during the pandemic among the individuals living in Brazil, 2020.

Factors		OR (95%CI)	*p*-Value	aOR (95%CI)	*p*-Value
Lives in the Metropolitan Area	Yes	0.927 (0.746–1.151)	0.493		
	No	1.00			
Age group	18–25 years old	1.00			
	26–39 years old	0.936 (0.740–1.185)	0.585		
	40+ years old	1.572 (1.176–2.101)	0.002		
Schooling level	High School	1.00			
	Higher Education	2.171 (0.618–7.629)	0.227		
	Postgraduate Degree	2.509 (0.727–8.656)	0.145		
In isolation	Yes	1.00			
	Partially	0.637 (0.325–1.249)	0.189		
	No	1.356 (1.096–1.677)	0.005		
Impact	A little	1.730 (1.273–2.350)	<0.001	1.375 (0.959–1.972)	0.015
	To some extent	0.998 (0.726–1.373)	0.991	0.822 (0.568–1.189)	0.683
	Very much	1.00		1.00	
Type of partners	Casual	1.837 (1.388–2.433)	<0.001		
	Stable and occasional	0.749 (0.588–0.953)	0.019		
	Stable	1.00			
Lives with partner	Yes	1.00			
	No	1.085 (0.814–1.445)	0.579		
Uses dating apps	Yes	1.190 (0.936–1.513)	0.155		
	No	1.00			
Uses Facebook to find partners	Yes	0.757 (0.623–0.922)	0.006		
	No	1.00			
Uses WhatsApp groups	Yes	0.603 (0.400–0.909)	0.016		
	No	1.00			
Goes to bars to find partners	Yes	1.013 (0.709–1.446)	0.944		
	No	1.00			
Does not look for partners during the pandemic	Yes	0.678 (0.528–0.872)	0.002		
	No	1.00			
During the pandemic, the frequency of your sexual practices	Did not change	1.00			
	Was reduced	1.066 (0.814–1.396)	0.643		
	Increased	1.085 (0.784–1.500)	0.624		
During the pandemic, your number of partners	Did not change	1.00			
	Was reduced	0.799 (0.649–0.985)	0.035		
	Increased	3.312 (2.381–4.607)	<0.001		
Practiced group sex during the pandemic	Yes	1.174 (0.867–1.589)	0.299	5.928 (4.106–8.559)	<0.001
	No	1.00		1.00	
Practiced chemsex during the pandemic	Yes	1.472 (1.208–1.792)	<0.001	1.764 (1.291–2.412)	<0.001
	No	1.00		1.00	
Multiple partners (3 or more) during the pandemic	Yes	4.676 (3.811–5.739)	<0.001	19.131 (13.642–26.828)	<0.001
	No	1.00		1.00	
Already presented COVID-19 symptoms	Yes	1.00			
	No	2.238 (1.848–2.712)	<0.001		
Was already tested for COVID-19	Yes	1.00			
	No	1.605 (1.100–2.342)	0.014		
Alcohol consumption during the pandemic	Did not change	1.00			
	Was reduced	1.150 (0.891–1.482)	0.283		
	Increased	1.133 (0.872–1.473)	0.351		
Do you know your HIV status?	Yes	1.00			
	No	1.130 (0.865–1.147)	0.370		
Tested for HIV in the last 12 months	Yes	1.025 (0.848–1.238)	0.800	1.336 (1.063–1.680)	0.013
	No	1.00		1.00	
Previous history of at-risk sexual exposure	Yes	4.104 (3.129–5.384)	<0.001	8.861 (6.454–12.166)	<0.001
	No	1.00		1.00	

OR: odds ratio, aOR: adjusted odds ratio, CI: confidence interval, ROC curve: 0.794 (<0.001); sensitivity: 79.3%; specificity: 66.3%, Nagelkerke R square: 0.333.

**Table 4:** Bivariate and multivariate analysis considering the factors associated with at-risk sexual exposure during the pandemic among the individuals living in Portugal, 2020.

Factors		OR (95%CI)	*p*-Value	aOR (95%CI)	*p*-Value
Lives in the Metropolitan Area	Yes	1.518 (0.859–2.680)	0.151	5.673 (2.511–12.814)	<0.001
	No	1.00		1.00	
Age group	18–25 years old	1.00			
	26–39 years old	0.945 (0.673–1.326)	0.743		
	40+ years old	0.871 (0.548–1.385)	0.560		
Schooling level	High School	1.00			
	Higher Education	0.843 (0.333–2.131)	0.717		
	Postgraduate Degree	2.273 (1.087–4.753)	0.029		
In isolation	Yes	1.00			
	Partially	1.496 (1.043–2.147)	0.029		
	No	2.406 (1.094–5.294)	0.029		
Impact	A little	3.106 (1.735–5.560)	0.071		
	To some extent	1.733 (0.955–3.145)	<0.001		
	Very much	1.00			
Type of partners	Casual	1.289 (0.916–1.814)	0.145		
	Stable and occasional	0.629 (0.356–1.113)	0.111		
	Stable	1.00			
Lives with partner	Yes	1.00		1.00	
	No	3.513 (2.020–6.110)	<0.001	5.556 (2.857–10.803)	<0.001
Uses dating apps	Yes	1.198 (0.848–1.693)	0.305		
	No	1.00			
Uses Facebook to find partners	Yes	0.980 (0.721–1.332)	0.898		
	No	1.00			
Uses WhatsApp groups	Yes	0.465 (0.259–0.835)	0.010		
	No	1.00			
Goes to bars to find partners	Yes	0.755 (0.440–1.296)	0.308		
	No	1.00			
Does not look for partners during the pandemic	Yes	0.665 (0.415–1.067)	0.091		
	No	1.00			
During the pandemic, the frequency of your sexual practices	Did not change	1.00			
	Was reduced	0.536 (0.268–1.073)	0.984		
	Increased	1.006 (0.564–1.795)	0.078		
During the pandemic, your number of partners	Did not change	1.00			
	Was reduced	0.425 (0.300–0.604)	<0.001		
	Increased	1.625 (1.147–3.952)	0.989		
Practiced group sex during the pandemic	Yes	57.244 (24.440–134.076)	<0.001		
	No	1.00			
Practiced chemsex during the pandemic	Yes	9.200 (5.918–14.302)	<0.001		
	No	1.00			
Had multiple partners (3 or more) during the pandemic	Yes	19.851 (13.588–29.003)	<0.001	17.808 (11.099–28.571)	<0.001
	No	1.00		1.00	
Already presented COVID-19 symptoms	Yes	1.00		1.00	
	No	2.521 (1.847–3.443)	<0.001	5.416 (3.293–8.909)	<0.001
Was already tested for COVID-19	Yes	1.541 (1.055–2.251)	0.025		
	No	1.00			
Alcohol consumption during the pandemic	Did not change	1.00			
	Was reduced	0.770 (0.493–1.202)	0.250		
	Increased	1.080 (0.716–1.641)	0.704		
Do you know your HIV status?	Yes	1.00			
	No	0.680 (0.454–1.020)	0.062		
Tested for HIV in the last 12 months	Yes	1.407 (1.031–1.921)	0.031		
	No	1.00			
Previous history of at-risk sexual exposure	Yes	1.656 (1.186–2.313)	0.003		
	No	1.00			

OR: odds ratio, aOR: adjusted odds ratio, CI: confidence interval, ROC curve: 0.876 (<0.001); sensitivity: 92.6%; specificity: 73.5%, Nagelkerke R square: 0.476.

## Data Availability

Additional data are available as long as the request is made to the corresponding author.
